# ZEB1 limits adenoviral infectability by transcriptionally repressing the Coxsackie virus and Adenovirus Receptor

**DOI:** 10.1186/1476-4598-10-91

**Published:** 2011-07-27

**Authors:** Markus D Lacher, Marisa Shiina, Peter Chang, Debora Keller, Maarit I Tiirikainen, W Michael Korn

**Affiliations:** 1 Dept. of Medicine, Division of Gastroenterology, University of California, San Francisco, CA, USA; 2 Helen Diller Family Comprehensive Cancer Center, University of California, San Francisco, CA, USA; 3 Current Address: BioTime, Inc., 1301 Harbor Bay Parkway, Suite 100, Alameda, 94502, CA, USA; 4 Current Address: Cardiac Department National University Heart Centre Singapore, NUHS Tower Block, Level 9, 1E Kent Ridge Road, Singapore, 119228, Republic of Singapore; 5 Current Address: Swiss Institute for Experimental Cancer Research (ISREC), Swiss Federal Institute of Technology (EPFL) School of Life Sciences, Lausanne, 1015, Switzerland; 6 Current Address: University of Hawaii Cancer Center, University of Hawaii, Honolulu, HI, USA; 7 Dept. of Medicine, Division of Medical Oncology, University of California, San Francisco, CA, USA

**Keywords:** ZEB1, EMT, MET, TGF-β, adenovirus, cancer

## Abstract

**Background:**

We have previously reported that RAS-MEK (Cancer Res. 2003 May 1;63(9):2088-95) and TGF-β (Cancer Res. 2006 Feb 1;66(3):1648-57) signaling negatively regulate coxsackie virus and adenovirus receptor (CAR) cell-surface expression and adenovirus uptake. In the case of TGF-β, down-regulation of CAR occurred in context of epithelial-to-mesenchymal transition (EMT), a process associated with transcriptional repression of E-cadherin by, for instance, the E2 box-binding factors Snail, Slug, SIP1 or ZEB1. While EMT is crucial in embryonic development, it has been proposed to contribute to the formation of invasive and metastatic carcinomas by reducing cell-cell contacts and increasing cell migration.

**Results:**

Here, we show that ZEB1 represses CAR expression in both PANC-1 (pancreatic) and MDA-MB-231 (breast) human cancer cells. We demonstrate that ZEB1 physically associates with at least one of two closely spaced and conserved E2 boxes within the minimal CAR promoter here defined as genomic region -291 to -1 relative to the translational start ATG. In agreement with ZEB1's established role as a negative regulator of the epithelial phenotype, silencing its expression in MDA-MB-231 cells induced a partial Mesenchymal-to-Epithelial Transition (MET) characterized by increased levels of E-cadherin and CAR, and decreased expression of fibronectin. Conversely, knockdown of ZEB1 in PANC-1 cells antagonized both the TGF-β-induced down-regulation of E-cadherin and CAR and the reduction of adenovirus uptake. Interestingly, even though ZEB1 clearly contributes to the TGF-β-induced mesenchymal phenotype of PANC-1 cells, TGF-β did not seem to affect ZEB1's protein levels or subcellular localization. These findings suggest that TGF-β may inhibit CAR expression by regulating factor(s) that cooperate with ZEB1 to repress the CAR promoter, rather than by regulating ZEB1 expression levels. In addition to the negative E2 box-mediated regulation the minimal CAR promoter is positively regulated through conserved *ETS *and *CRE *elements.

**Conclusions:**

This report provides evidence that inhibition of ZEB1 may improve adenovirus uptake of cancer cells that have undergone EMT and for which ZEB1 is necessary to maintain the mesenchymal phenotype. Targeting of ZEB1 may reverse some aspects of EMT including the down-regulation of CAR.

## Background

The coxsackie virus and adenovirus receptor (CAR), encoded by the *CXADR *gene, is localized at the apicolateral/basolateral surface of polarized epithelial cells and serves as a component of tight junctions, thus participating in the sealing of the epithelial layer. In addition to its basolateral localization, recently, an apically localized isoform (CAR^Ex8^) was described which may be responsible for initiation of respiratory adenoviral infections [1]. Furthermore, CAR regulates cardiac conductance, as demonstrated in a mouse model in which heart-specific inducible CAR knockout resulted in impaired electrical conductance between atrium and ventricle [2].

CAR is the primary receptor for adenovirus serotypes 2 and 5 [3] and thus a likely determining factor for the efficacy of adenovirus-based cancer therapy. A number of mechanisms by which CAR expression is regulated have been described, but our understanding of how to manipulate CAR expression levels in cancer is incomplete [4-11]. Learning the molecular machinery regulating CAR expression could set the stage for pharmacological interventions aimed at achieving high cell surface CAR levels to maximize virus uptake.

We previously identified RAS-MEK [5] and TGF-β signaling [9] as negative regulators of CAR expression in cancer cell lines. Down-regulation of CAR through TGF-β occurred in the context of epithelial-to-mesenchymal transition (EMT), a process that refers to the formation of mesenchymal cells from epithelial cells without the involvement of stem cells. During EMT, both tight junctions at apicolateral surfaces containing CAR, and more basolateral adherens junctions containing E-cadherin are disrupted, and cells acquire a motile phenotype. EMT has evolved as an important developmental program. However, inappropriate activation is linked to pathological conditions such as fibrosis and cancer [12]. In the case of cancer, EMT may contribute to the formation of invasive and metastatic carcinomas by reducing cell-cell contacts and increasing cell migration [13-15]. Additionally, the EMT-associated reduction of cell surface CAR likely makes advanced malignancies with already poor prognosis less responsive to treatment with oncolytic adenoviruses [5,9].

One of the most prominent inducers of EMT is TGF-β. It is postulated that TGF-β inhibits cell cycle progression, but alters the tumor microenvironment, promotes EMT, immunosuppression and angiogenesis in advanced malignancies, thus playing both tumor suppressive and oncogenic roles during multistage carcinogenesis [16-22]. The switch from tumor suppressor to oncogene may occur upon loss of the cytostatic arm of the TGF-β pathway, for instance through genetic inactivation of tumor suppressive TGF-β downstream effectors such as p15INK4b, a cyclin-dependent kinase (CDK) inhibitor [20].

Mechanisms underlying TGF-β-induced EMT involve E2 box-binding transcriptional repressors, in particular Snail (*SNAI1*), Slug (*SNAI2*), SIP1 (*ZEB2*) and ZEB1 (*ZEB1*). These repressors target genes whose protein products are instrumental for the integrity of the epithelial phenotype [23-25]. Interestingly, in addition to regulating protein-encoding genes, ZEB1 and SIP1 are both targets and negative regulators of microRNA-200 (miR-200) family members. Depending on whether an extracellular stimulus up-regulates ZEB1 or SIP1, or raises miR-200 levels, the resulting positive feedback loop may stabilize either a mesenchymal (elevated ZEB1 and/or SIP1 activity) or an epithelial (increased miR-200 levels) state [26-28]. Furthermore, consistent with the proposed contribution of EMT to cancer progression, expression of E2 box-binding repressors has been observed in several malignancies [25,29-32].

The aim of this study was to examine the mechanism by which TGF-β down-regulates CAR. By investigating how RAS-MEK [5] and TGF-β signaling [9] impact on CAR expression, we noticed similar expression patterns for CAR and E-cadherin, suggesting common underlying regulatory mechanisms. We show here that for the regulation through TGF-β this is indeed the case. Both CAR and E-cadherin promoters are structurally conserved around two closely spaced E2 boxes. We provide evidence that ZEB1, which has previously been reported to repress E-cadherin expression [25,33-36], also down-regulates CAR. This study, in combination with the work of others [26,27,34,36], identifies ZEB1 as a potential therapeutic target for strategies aimed at improving uptake of therapeutic adenoviruses and preventing or reversing cancer-associated EMT processes while leaving the tumor suppressive functions of TGF-β unaffected. As our work was in progress, a report was published demonstrating that TGF-β may repress the mouse CAR promoter through Snail in combination with Smad3/4 [37]. Our data is consistent with a model in which both ZEB1 and Snail-Smad3/4 can simultaneously repress the human CAR promoter.

## Methods

Additional methods and further details including antibodies are provided in the Additional file 1.

### *In silico *analyses

**Figure 1:**
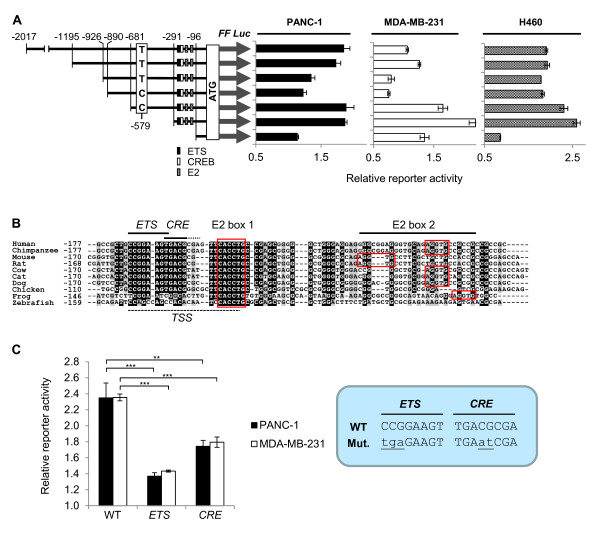
**Structure of the CAR promoter**. **A**. Several *CXADR *(CAR) upstream fragments were PCR-amplified from human genomic DNA and cloned into a firefly (FF) luciferase vector in endogenous constellation, i.e. without vector sequence between the CAR regulatory region, 5'-UTR and the translational start of the luciferase coding sequence. The resulting 5'-deletion series was transfected into PANC-1, MDA-MB-231 and H460 cells, in combination with pRL-SV40 (Promega) encoding renilla (RL) luciferase. Cells were lysed twenty-four hours post transfection, and promoter activities were measured with the Dual-Luciferase^® ^Reporter Assay System (Promega). Reporter activities are displayed as fold changes of the FF:RL luciferase RLU (relative light unit) ratios relative to the empty vector. **B**. Alignment of orthologous CAR promoter sequences and identification of conserved putative *ETS *and *CRE *sites [40-42,49]. A region in which mouse CAR transcripts are likely initiated is indicated (TSS) [56]. Human CAR transcripts may start at around 150 bp upstream of the translational start ATG [10]. **C**. Wild-type (WT), *ETS *and *CRE *mutant -291/-1 CAR promoter constructs were transfected into PANC-1 and MDA-MB-231 cells. Cell lysis and measurements of promoter activities were conducted as in A. Error bars represent standard deviations (biological triplicates). p < 0.001 (***), p < 0.01 (**) (Student's *t*-test: 1-tailed, equal variance).

Orthologous CAR upstream sequences were exported from the GenBank (http://www.ncbi.nlm.nih.gov) or Ensemble (http://www.ensembl.org) database according to the positions of the predicted translational start ATG (Additional file 1, Table S1). The sequence alignment was performed with CLUSTAL W 1.83 [38] with sub-sequences (-25 to +60 relative to the "C" of the CACCTG E2 box corresponding to E2 box 1 in Figure 1A and 1B) encoded within the -291/-1 region of the human *CXADR *(CAR) gene. Conserved nucleotides of the aligned sequences were shaded with BOXSHADE 3.21 (http://www.ch.embnet.org/software/BOX_form.html). A stretch of dog CAR sequence previously not available in public databases was sequenced at the University of California, San Francisco (UCSF) Helen Diller Family Comprehensive Cancer Center (HDFCCC) Genome Core (San Francisco, CA, USA) using PCR-amplified genomic DNA extracted from MDCK-Tetoff-SIP1 cells [39] as template. The obtained sequence was submitted to the GenBank database (NCBI, accession number EU744539). In Figure 1B highlighted *ETS *and cAMP responsive element (*CRE*) elements are represented by or are highly similar to TRANSFAC^® ^[40] consensus sequences [*ETS*: TRANSFAC^® ^acc. # R02153 (ETS1$CONS, SMGGAWGY), *CRE*: TRANSFAC^® ^acc. # R04110 (CREB$CONS_01, TGACGTMW), S = G or C, W = A or T, M = A or C, Y = C or T] and motifs in published reports (*CRE*, [41,42]).

### Cell lines

The human pancreatic cancer cell line PANC-1 [9], and the human breast cancer cell line MDA-MB-231 (gift from Dr. J. Gray, Lawrence Berkeley National Laboratory) were maintained in Dulbecco's Modified Eagle Medium (DMEM; UCSF Cell Culture Facility, San Francisco, CA, USA) supplemented with 10% Fetal Bovine Serum (FBS; Valley Biomedical, Inc.; Winchester, VA, USA) and 100 units/mL penicillin "G", 100 mcg/mL streptomycin SO_4 _(both UCSF Cell Culture Facility), and 5 microgram/mL Plasmocin™ (InvivoGen, San Diego, CA, USA). The human non-small cell lung cancer cell line H460 (gift from Dr. D. Jablons, UCSF) [43] was grown in RPMI-1640 (Gibco/Invitrogen, Carlsbad, CA, USA), supplemented with 10% FBS, penicillin, streptomycin and Plasmocin (all supplemented components as above).

### Constructs

Various CAR [promoter]-[5'-UTR] fragments were independently PCR-amplified from human genomic DNA and cloned into pGL3Ba-DESneo3N. The sequence between the translational ATG start codons of CAR and luciferase was removed by restriction digestion, followed by ethanol precipitation and re-ligation. Mutations at the E2 boxes, *ETS *and *CRE *motifs were introduced into the -291/-1 luciferase construct. Inducible Myc-tagged ZEB1 expression constructs were generated by replacing the mSIP1 coding sequence (cds) of pUHD10.3SIP1 [39] through PCR-amplified human *ZEB1 *cds. Primer sequences and cloning strategies are provided as supplemental information (Additional file 1).

### Immunofluorescence and F-actin staining

PANC-1 and MDA-MB-231 cells were grown on Lab-Tek™ Chamber Slides (Nalge Nunc/Thermo Fisher Scientific, Inc., Rockford, IL, USA) and treated with 5 ng/mL platelet-derived human TGF-β1 (R&D Systems, Minneapolis, MN, USA) for four days. For E-cadherin staining, cells were fixed with a 1:1 solution of methanol and acetone at -20°C, and unspecific epitopes were blocked with 3% bovine serum albumin (BSA) (Sigma-Aldrich, St. Louis, MO, USA). Then, cells were incubated for 1 hour with 2 microgram/mL of the mouse anti-E-cadherin antibody (clone HECD-1, Invitrogen). For F-actin and vimentin stainings, cells were fixed for 15 min. with IC Fixation Buffer (Invitrogen) and permeabilized for 5 min. with 0.1% Triton X-100 (Aqua Solutions, Deer Park, TX, USA). Then, unspecific epitopes were blocked with 3% BSA and cells were incubated for 1 hour with a 1:100 dilution of phalloidin conjugated to Texas Red^® ^(Invitrogen) or with a 1:100 dilution of the rabbit anti-vimentin antibody (D21H3, Cell Signaling Technology, Inc., Danvers, MA, USA). For E-cadherin and vimentin stainings secondary antibodies conjugated to Alexa Fluor^® ^488 (Molecular Probes/Invitrogen) were used. Nuclei were stained with DAPI, and samples mounted onto glass slides using Vectashield (Vector Lab, Burlingame, CA, USA). Immunofluorescence images were obtained using a Zeiss Imager Z2 microscope (Carl Zeiss, Jena, Germany) equipped with an AxioCam camera and processed with Axiovision software. Digital images were adjusted for contrast and brightness using Adobe Photoshop CS5.

### RNA interference

PANC-1 cells were pre-treated for two days with 5 ng/mL platelet-derived human TGF-β1 (R&D Systems), then, and two days later, siRNA-transfected by using Lipofectamine RNAiMax (Invitrogen). TGF-β treatment was continued through the first, until two days after the second transfection. MDA-MB-231 cells were similarly transfected, but not stimulated with ectopic TGF-β. Cell lysis for protein harvest, flow cytometric analysis of cell-surface CAR and adenovirus infections were carried out four days after the initial transfection. Abbreviations: UT, untransfected; Ctrl #1, siControl ON-TARGETplus Non-targeting siRNA #1 (Dharmacon/Thermo Fisher Scientific, Inc.); Ctrl #2, firefly luciferase-targeting siRNA; ZEB1 siRNA #1/#2, ZEB1-targeting siRNAs. Ctrl #2 and ZEB1 siRNA sequences are provided in Additional file 1 (Table S3) and were obtained by using the si*DESIGN*^® ^Center (Dharmacon/Thermo Fisher Scientific, Inc.). Detailed information is provided as supplemental information (Additional file 1).

### Expression analysis by real-time RT-PCR

Total RNA was extracted with the RNeasy kit (Qiagen, Valencia, CA, USA). Reverse-transcription and real-time PCR were carried out at the UCSF HDFCCC Genome Core with the primer/probe sequences listed in Additional file 1 (Table S3) and with Expression Assays (Applied Biosystems, Foster City, CA, USA) for *CDH1 *(E-cadherin, Hs00170423_m1), *ZEB1 *(Hs00611018_m1 or Hs00232783_m1), *ZEB2 *(SIP1, Hs00207691_m1), *SNAI1 *(Snail, Hs00195591_m1), *SNAI2 *(Slug, Hs00161904_m1) and *SERPINE1 *(PAI-1, Hs01126604_m1). Data were analyzed by relative quantitation [44].

### Immunoblotting and cell fractionation

Antibodies used include rabbit anti-phospho-Smad2 (Ser465/467, Cell Signaling Technology, Inc.), goat anti-ZEB1 (E-20, Santa Cruz Biotechnology, Inc., Santa Cruz, CA, USA), mouse anti-β-tubulin (Sigma-Aldrich), mouse anti-PARP (clone C2-10, Pharmingen/BD Biosciences, San Jose, CA, USA), mouse anti-GAPDH-Peroxidase Conjugate (Sigma-Aldrich), and mouse anti-Myc Tag (clone 4A6; Upstate/Millipore, Charlottesville, VA, USA). Cell fractionation was carried out via the NE-PER^® ^Nuclear and Cytoplasmic Extraction Reagents kit (Pierce/Thermo Fisher Scientific, Inc.). A description of the Western blot procedure and further antibody references are provided elsewhere [9].

### Luciferase reporter assays

All transfections involving CAR promoter constructs were carried out by using FuGENE HD (Roche, Indianapolis, IN, USA) (3 microliter per 1 microgram of DNA), and included co-transfection of the *renilla *luciferase-encoding pRL-SV40 plasmid (Promega, Madison, WI, USA) for normalization. Cells were subconfluent at the time of transfection. For the identification of the CAR promoter, cells were grown in 24-well plates and transfected with 750 nanogram of the pGL3Ba-DESneo3N reporter plasmids in combination with 10 nanogram pRL-SV40. To transfect equimolar amounts of each CAR promoter construct of the CAR upstream 5'-deletion series, plasmid size differences were compensated by co-transfection with the pGL3Ba-DESneo3N-EmVec empty vector plasmid. For the characterization of the *ETS *and *CRE *elements, cells were grown in 6-well plates and transfected with 3 microgram of wild-type, *ETS *or *CRE *element-mutated -291/-1 luciferase construct in combination with 50 nanogram pRL-SV40. For the characterization of the E2 boxes as binding sites for ZEB1, cells were grown in 24-well plates and transfected with 500 nanogram of wild-type and E2 box-mutated -291/-1 luciferase construct, 125 nanogram pRevTet-Off (Clontech Laboratories, Inc./Takara Bio, Inc., Otsu, Shiga, Japan), and 375 nanogram pTRE-6Myc-deltaATG-hZEB1 in combination with 10 nanogram pRL-SV40. 4-6 hours post transfection, the transfection medium was removed, and around 1.5-2 hours later, stimulation with 2 microgram/mL doxycyline hyclate (Sigma-Aldrich) was begun. Cells were lysed twenty-four (to define the minimal CAR promoter and to characterize *ETS *and *CRE *elements) or forty-eight (to assess effects of Myc-ZEB1 on the WT and mutant CAR promoter) hours post transfection with Passive Lysis Buffer (Promega). Reporter activities were measured with the Dual-Luciferase^® ^Reporter Assay System (Promega).

### Biotinylated Oligonucleotide Precipitation Assay

One day after seeding 3 × 10^6 ^PANC-1 cells per 10 cm-dish, cells were transiently co-transfected with pRevTet-Off (4.0 microgram) (Clontech Laboratories, Inc./Takara Bio, Inc.) in combination with pTRE-6Myc-deltaATG-hZEB1 (12.0 microgram) by using FuGENE HD (Roche) (3 microliter per 1 microgram of DNA). Control lysates were made from PANC-1 cells seeded at a density of 5 × 10^5 ^cells per well (6-well plate) and transfected with the same plasmids. Four hours post transfection, transfection medium was replaced by antibiotic-containing full medium. Six hours post transfection, medium was again replaced by full medium with (to repress ZEB1) or without (to induce ZEB1) 2 microgram/mL doxycycline hyclate (Sigma-Aldrich). Forty-eight hours after transfection, oligonucleotide precipitations were carried out following a modified version of the procedure described by others [39,45]. ZEB1 was detected with the mouse monoclonal anti-Myc Tag clone 4A6 (Upstate/Millipore) antibody at 1 microgram/mL. Detailed information is provided as supplemental information (Additional file 1).

### Chromatin Immunoprecipitation

PANC-1 cells were transiently transfected with pTRE-6Myc-deltaATG-hZEB1 in combination with pRevTet-Off (Clontech) using FuGENE HD (Roche). For the control sample, six hours after addition of the plasmid DNA to the cells, expression of Myc-ZEB1 was suppressed with 2 microgram/mL doxycyline hyclate (Sigma-Aldrich). The next day, cells of both control and experimental samples were stimulated with 5 ng/mL platelet-derived human TGF-β1 (R&D Systems). Forty-eight hours after transfection, chromatin was cross-linked with paraformaldehyde and subjected to Chromatin Immunoprecipitation (ChIP) at the University of California at Davis (UC Davis) Genome Center (CA, USA), following a protocol developed by the Farnham laboratory (UC Davis, Davis, CA, USA) (http://www.genomecenter.ucdavis.edu/farnham/pdf/FarnhamLabChIP%20Protocol.pdf). In short, samples were sonicated using a BioRuptor™ Sonicator (Diagenode, Inc.; Sparta, NJ, USA), DNA was precipitated with an anti-Myc Tag antibody (clone 4A6; Upstate/Millipore), and SYBR Green I real-time PCR with the precipitated DNA as template was conducted using the iQ™ SYBR^® ^Green Supermix (Bio-Rad Laboratories, Hercules, CA, USA) using CAR promoter-specific primers (Additional file 1, Table S3).

### Adenovirus infections

Following a four-day siRNA treatment period, PANC-1 cells were infected with 300 microliter/well (12-well plates) Ad-GFP [9] diluted in DMEM (UCSF Cell Culture Facility) supplemented with 2% FBS (Valley Biomedical, Inc.) at a Multiplicity Of Infection (MOI) of 200. Ninety minutes post-infection, virus was replaced by regular growth medium. Twenty-four hours post-infection, Ad-GFP uptake was analyzed by both flow cytometry (GFP intensities) and real-time PCR (virus copy numbers). For the latter approach genomic/adenoviral DNA was first extracted with the DNeasy^® ^Blood & Tissue kit (Qiagen) and then subjected to ethanol precipitation to potentially improve DNA quality. Relative virus copy numbers were determined at the UCSF HDFCCC Genome Core by TaqMan PCR amplification of the adenovirus fiber gene (primer/probe sequences shown in Additional file 1, Table S3) normalized to genomic DNA amplified with a pool of primers for D1S2868, D2S385, D4S1605, D5S643, D10S586, and D11S1315 [46]. Data were analyzed by relative quantitation [44].

### Flow cytometry

Live cells were stained with an anti-CAR-phycoerythrin (PE) antibody (E1-1, mouse monoclonal; Santa Cruz Biotechnology, Inc.) or PE-conjugated control IgG-PE (mouse monoclonal IgG1 κ, Pharmingen/BD Biosciences) while rotating for 60 minutes at 4°C. Cells were then washed and resuspended in 1 micromolar TO-PRO^®^-3 iodide (TP3, for exclusion of dead cells) (Invitrogen) in PBS supplemented with 5% FBS, and analyzed by flow cytometry using FACSCalibur (BD Biosciences) or Accuri C6 (Accuri Cytometers, Inc., Ann Arbor, MI, USA/BD, Franklin Lakes, NJ, USA) flow cytometers. Cell-surface CAR was detected in the FL2 channel, non-viable cells, stained by TP3 and detected in the FL4 channel, were excluded. For the analysis of live Ad-GFP infected cells, GFP was detected in the FL1 channel. TP3-positive cells were excluded. Data analysis was carried out with Cyflogic™ software (CyFlo Ltd, Turku, Finland). Detailed information is provided as supplemental information (Additional file 1).

## Results

### Defining the CAR promoter

TGF-β down-regulates CAR mRNA and protein levels [9]. Since neither mRNA nor protein stability appeared to be affected by TGF-β [9], regulation of CAR expression likely occurs at the promoter level. Bowles *et al*. reported that the locus of the functional human CAR gene (*CXADR*) is on chromosome 21, 21q11.2 [47]. However, even though 21q11 harbors CAR sequence, this locus encodes a CAR pseudogene lacking introns. The functional human CAR gene is located on 21q21.1 (Entrez Gene, Gene ID: 1525, NCBI).

To experimentally determine the CAR promoter region we cloned several fragments of CAR upstream sequence as a 5'-deletion series into pGL3Ba-DESneo3N (a luciferase reporter vector we engineered allowing recombination-based cloning of [promoter]-5'-UTR fragments in endogenous constellation, i.e. without vector sequence between the regulatory fragments and the luciferase coding sequence (Figure 1A). To identify genomic regions involved in the regulation of CAR expression, we transfected the 5'-deletion series into PANC-1 (human pancreatic cancer), H460 (human non-small cell lung cancer), and MDA-MB-231 (human breast cancer) cells. In all cell lines, reporter activities were higher for the genomic fragments -2017/-1, -1195/-1, -681/-1, -291/-1 than for -926/-1, and -890/-1 (Figure 1A). This may suggest that silencer elements are present between -1194 and -682, and that positive regulatory elements further upstream override this negative regulation. In all cell lines, maximal promoter activity was measured with the -291/-1 construct, whereas the -96/-1 fragment was only minimally active. Therefore, the CAR core promoter, which interacts with the DNA polymerase II complex, and the adjacent proximal promoter [48], are located within -291 and -1 relative to the translational start ATG. This is in agreement with a previous report by Pong *et al*. illustrating that CAR transcription is likely initiated at around -150 relative to the ATG [10].

Since each promoter/5'-UTR fragment was individually PCR-amplified we were able to identify a single nucleotide polymorphism (SNP) at position -579, with the base being either thymine (in the -2017/-1, -1195/-1, -926/-1 fragments) or cytosine (-890/-1 and -681/-1 fragments). It is unlikely that this SNP influences CAR expression, since the reporter activities of the -926/-1 and the -890/-1 fragments, which differ only in 36 bp, are very similar, despite the polymorphic difference (Figure 1A and data not shown).

By aligning CAR upstream sequences from diverse species ranging from zebrafish to man, several conserved elements were recognized within the -291/-1 fragment: putative binding sites for ETS transcription factors and for c-AMP responsive element (*CRE*) binding protein (CREB) [41,42,49], as well as two closely spaced E2 boxes (Figure 1B). The latter elements are particularly interesting since they are located in a similar genetic context than the E2 boxes in the human E-cadherin promoter to which E2 box-binding repressors such as SIP1 [23,33,35,39] and ZEB1 [25,33-36] bind. To investigate whether the *ETS *and *CRE *elements are biologically relevant, we transiently transfected PANC-1 and MDA-MB-231 cells with *ETS *or *CRE *mutant -291/-1 luciferase constructs. Inactivation of either motif reduced CAR promoter activity, suggesting that both ETS and CREB factors may induce CAR expression (Figure 1C).

### Down-regulation of CAR in TGF-β-induced EMT

**Figure 2:**
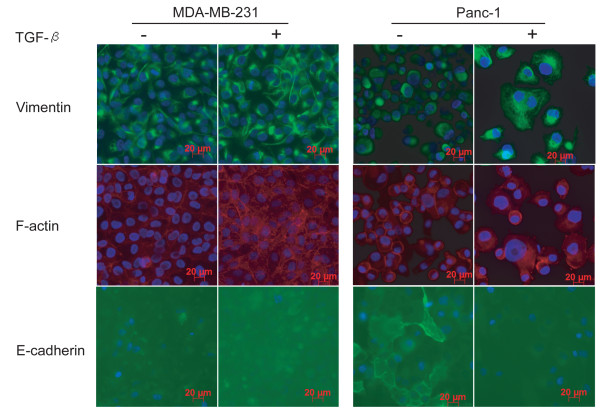
**Effect of TGF-β on EMT markers**. MDA-MB-231 and PANC-1 cells were stimulated with TGF-β for four days, then assessed for E-cadherin and vimentin expression/localization by immunofluorescence, or were stained with phalloidin conjugated to Texas Red^® ^to visualize F-actin. Cell surface expression of E-cadherin is a hallmark of an epithelial phenotype. Vimentin intermediate filaments and F-actin are features of mesenchymal cells.

**Figure 3:**
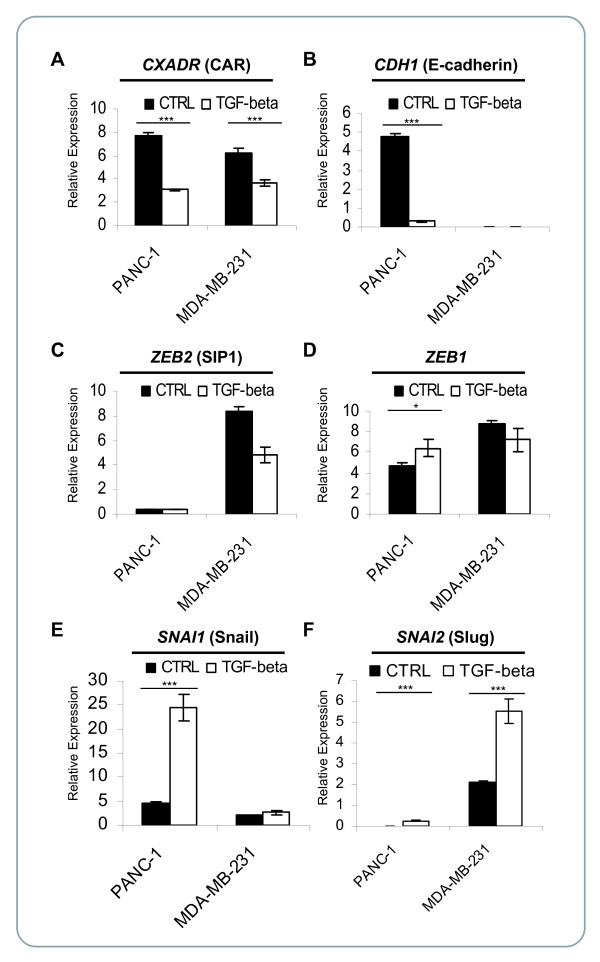
**E2 box-binding repressor expression upon TGF-β stimulation**. PANC-1 and MDA-MB-231 cells were treated with recombinant human TGF-β1 for four days. mRNA expression of *CXADR *(CAR) (**A**), *CDH1 *(E-cadherin) (**B**), *ZEB2 *(SIP1) (**C**), *ZEB1 *(**D**), *SNAI1 *(Snail) (**E**), and *SNAI2 *(Slug) (**F**) was measured by TaqMan real-time PCR. Expression data were normalized to *H3F3A *(H3 histone, family 3A) mRNA levels (arbitrary units). **A**-**F**. Error bars represent standard deviations (biological triplicates). p < 0.001 (***), p < 0.01 (**), p < 0.05 (*) (Student's *t*-test: 1-tailed, equal variance). Absence of * indicates p ≥ 0.05, or *decrease *of E2 box-binding repressor expression upon TGF-β stimulation.

The presence of the dual E2 box motif in the CAR promoter suggests that SIP1 and/or ZEB1 repress(es) CAR expression upon TGF-β treatment in cells undergoing EMT. If true, SIP1 and/or ZEB1 expression may be stimulated by TGF-β. We chose PANC-1 cells as an EMT model in this study as these cells are known to undergo TGF-β-induced EMT [9,50]. In agreement, untreated cells stained positive for cell surface E-cadherin but not for vimentin intermediate filaments or F-actin, thus demonstrating epithelial characteristics (Figure 2). Conversely, TGF-β induced an EMT process in PANC-1 cells as shown by lack of E-cadherin staining. In contrast, MDA-MB-231 cells did not express cell surface E-cadherin, but strongly stained positive for vimentin filaments or F-actin, thus demonstrating mesenchymal features (Figure 2). To address whether SIP1 and/or ZEB1 may affect CAR expression upon TGF-β stimulation, we measured their mRNA levels in PANC-1 and MDA-MB-231 cells. In agreement with the data obtained by immunofluorescence (Figure 2), MDA-MB-231 cells demonstrated mesenchymal features (lack of E-cadherin expression and high SIP1 and ZEB1 levels) (Figure 3B-D). It is of note that the cells used in this study are morphologically markedly different and may proliferate faster than MDA-MB-231 cells from the American Type Culture Collection (ATCC, Manassas, VA), and likely represent a derivative of the cell line. In agreement with our previous report, in PANC-1 cells, both CAR and E-cadherin mRNA levels were reduced as consequence of TGF-β treatment (Figure 3A and 3B) [9], while ZEB1 expression was modestly stimulated (Figure 3D). Despite the presence of a dual E2 box sequence in the CAR promoter single E2 box-binding repressors, such as Snail and Slug, may regulate CAR expression upon TGF-β stimulation. Indeed, PANC-1 cells responded to TGF-β stimulation with increased Snail expression (Figure 3E). This data is consistent with a recent report demonstrating that Snail-Smad3/4 is a physiological regulator of CAR in murine cells [37]. In addition to Snail, also Slug mRNA levels increased in PANC-1 cells following addition of TGF-β. However, as they remained low, Slug is not likely a regulator of CAR in these cells. Interestingly, despite their mesenchymal features, MDA-MB-231 cells expressed relatively high CAR levels (Figure 3A), and, similarly to PANC-1 cells, also down-regulated CAR upon TGF-β treatment. However, in MDA-MB-231 cells, TGF-β stimulated Slug expression, suggesting that in this cell line Slug potentially inhibits CAR expression (Figure 3F).

### E2 box-dependent repression of the human CAR promoter by ectopic ZEB1

**Figure 4:**
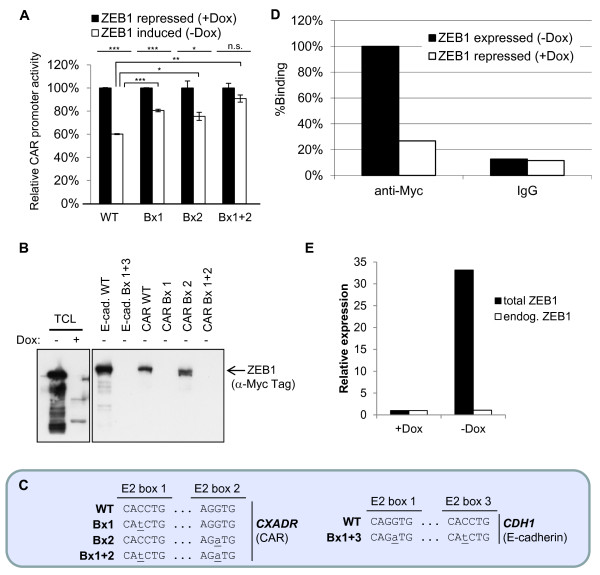
**E2 box-dependent repression of the CAR promoter and binding of ZEB1 to CAR promoter oligonucleotides and chromatin**. **A**. PANC-1 cells were transfected with CAR promoter/firefly (FF) luciferase constructs (-291/-1) in combination with pRL-SV40 (Promega) encoding renilla (RL) luciferase, an inducible Myc-ZEB1 expression construct, and a "Tet-OFF" plasmid allowing induction of *ZEB1 *by absence (-Dox), and repression by addition (+Dox) of doxycycline to the culture medium. Cells were lysed forty-eight hours post transfection, and promoter activities were measured with the Dual-Luciferase^® ^Reporter Assay System (Promega). Reporter activities are displayed as fold changes of the FF:RL luciferase RLU ratios relative to the empty vector. Error bars represent standard deviations (biological duplicates). **B**. In PANC-1 cells ectopically expressed Myc-tagged ZEB1 was precipitated with streptavidin-agarose resin and biotinlyated E-cadherin or CAR promoter oligonucleotides, and then subjected to immunoblotting and detection with an anti-Myc tag antibody. **C**. Mutations at the E2 boxes in the constructs transfected in A, and in the oligonucleotides used to precipitate ZEB1 (B). E-cadherin promoter mutations are described [39]. **D**. ChIP assay conducted with PANC-1 cells transiently transfeced with Myc-ZEB1 and stimulated with TGF-β. Myc-ZEB1 was precipitated with an anti-Myc Tag antibody. Co-precipitated DNA was amplified with CAR promoter-specific primers flanking E2 boxes 1 and 2. Shown are SYBR Green I real-time PCR data, normalized to input DNA (prior to precipitation with anti-Myc Tag antibody or control IgG). **E**. SYBR Green real-time PCR demonstrating overexpression of the total ZEB1 levels in the experiment shown in D. Abbreviations: TCL, total cell lysate; Dox, doxycyline; WT, wild-type; Bx 1, E2 box 1; Bx 2, E2 box 2; Bx 1+2, E2 boxes 1+2; Bx1+3, E2 boxes 1+3 ([39]). p < 0.001 (***), p < 0.01 (**), p < 0.05 (*), n.s.: not significant (Student's *t*-test: 1-tailed, equal variance).

A recent study indicates that CAR may be transcriptionally repressed by Snail-Smad3/4 in TGF-β stimulated murine epithelial cells [37]. However, microarray data suggests that siRNA-mediated knockdown of ZEB1 in human MDA-MB-231 cells may increase CAR mRNA levels [34]. Given the above described orthologously conserved nature of the E2 boxes in the CAR promoter, we hypothesized that the suggested repression of CAR is mediated by ZEB1 by directly repressing the CAR promoter at the E2 boxes, and is not an indirect consequence of the MET induced by the knockdown of ZEB1. To test this hypothesis, we co-transfected PANC-1 cells with an inducible Myc-tagged human ZEB1 expression plasmid, in combination with wild-type or E2 box-mutant CAR promoter reporter constructs. Induction of ZEB1 was performed in the context of a "Tet-OFF" system, in which the presence of doxycycline repressed ZEB1 expression, and carried out as a "dual luciferase" approach in which firefly (FF) luciferase was driven off the CAR promoter, and renilla (RL) luciferase was expressed through an SV40 promoter. While induction of ZEB1 repressed the wild-type CAR promoter, it also repressed the single E2 box-mutant promoters (Bx1, Bx2), although to a lesser degree. Repression of the CAR promoter was further reduced when both E2 boxes (Bx1+2) were inactivated. Importantly, compared to the wild-type promoter, all mutations resulted in significantly (p < 0.05) higher relative promoter activities in the presence of ZEB1 suggesting that ZEB1 indeed represses the CAR promoter E2 box-dependently (Figure 4A). It is important to note that a determination of the exact percentage of repression appeared not possible with the chosen dual luciferase approach, as various CAR promoter-independent factors affected the expression of both FF and RL luciferase. However, when correcting for such parameters mathematically (data not shown), several types of adjustment revealed stronger repression of the wild-type compared to the dual E2 box-mutant (Bx1+2) CAR promoter.

The presence of the dual E2 box motif suggests that, in addition to ZEB1, also SIP1 may repress the CAR promoter. Indeed, overexpression of Myc-tagged SIP1 [39] repressed CAR promoter activity E2 box-dependently (data not shown). However, since TGF-β neither increased SIP1 mRNA expression, nor are the SIP1 mRNA levels high in PANC-1 cells (Figure 3C) SIP1 is unlikely the main regulator of CAR in TGF-β-mediated EMT in our PANC-1 system.

### ZEB1 binds to the CAR promoter

To determine whether ZEB1 indeed physically binds to the E2 boxes in the CAR promoter, we overexpressed Myc-tagged human ZEB1 in PANC-1 cells and incubated the cell extracts with biotinylated oligonucleotides composed of a region of the CAR promoter containing the two E2 boxes (Additional file 1, Table S3). A similar strategy was used to elegantly demonstrate binding of SIP1 to the E-cadherin promoter [39]. Following pull-down with streptavidin-conjugated agarose resin, Myc-ZEB1 was detected by conventional Western blotting with an anti-Myc tag antibody. A strong signal was obtained with the oligonucleotides representing both wild-type and E2 box 2-mutant CAR promoter sequence. A mutation in either only E2 box 1 or in both E2 boxes prevented binding of ZEB1 to the oligonucleotides (Figure 4B). We conducted the same assay with Myc-tagged SIP1 [39] and, interestingly, observed a similar binding pattern (data not shown). However, as outlined above, SIP1 is unlikely the main repressor of CAR in TGF-β-mediated EMT in PANC-1 cells. Taken together, our data indicate that ZEB1 interacts with E2 box 1 but not with E2 box 2 (see Figure 1A for the location of E2 box 1 and 2 within the CAR promoter). It is conceivable that ZEB1 might still require both E2 boxes in the CAR promoter for binding, but the point mutation in E2 box 2 was insufficient to prevent binding (Figure 4C).

To ascertain whether ZEB1 also binds to the chromosomal CAR promoter in PANC-1 cells stimulated with TGF-β, a Chromatin Immunoprecipitation (ChIP) assay was conducted with cells transiently transfected with inducible Myc-ZEB1. As demonstrated in Figure 4D, precipitation of CAR DNA with an anti-Myc Tag antibody was apparent when Myc-ZEB1 was induced, suggesting binding of ZEB1 to genomic CAR promoter sequence. Nevertheless, some binding was also observed when Myc-ZEB1 was repressed (Figure 4D). However, this latter effect is likely due to leakiness of the system allowing some Myc-ZEB1 expression even in the presence of the repressor (doxycycline) (Figure 4E and Additional file 1, Fig. S1). As determined from sample aliquots removed prior to crosslinking, total ZEB1 mRNA levels were approximately 30 fold higher in the ChIP experiment following induction of Myc-ZEB1 expression by absence of doxycycline (Figure 4E).

### ZEB1 represses CAR in mesenchymal cells

**Figure 5:**
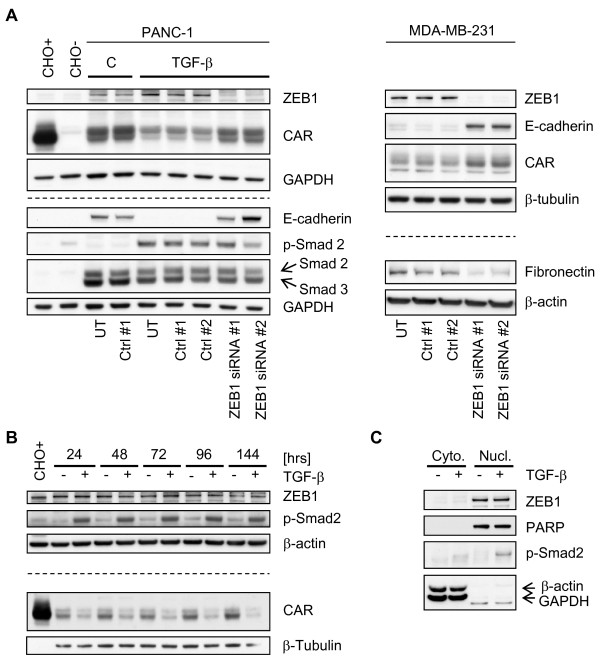
**ZEB1 promotes EMT**. **A-C**. Immunoblots. PANC-1 cells were pre-treated with TGF-β1 for two days and then transfected twice (day 0 and day 2) with ZEB1 siRNAs in the continued presence of TGF-β1. Four days after the initial transfection, cells were harvested. **A**. By up-regulating epithelial proteins such as E-cadherin and CAR, knockdown of ZEB1 antagonizes TGF-β-induced EMT in PANC-1 cells. Similarly, silencing of ZEB1 expression in MDA-MB-231 cells up-regulates E-cadherin and CAR, and down-regulates the mesenchymal marker fibronectin. **B**. PANC-1 cells were treated with TGF-β1, and harvested at the indicated time-points for analysis of the total protein fractions. **C**. PANC-1 cells were treated with TGF-β1 and subjected to cell fractionation. Abbreviations: C, TGF-β1 solvent control [4 mM HCl/0.1% (v/w) BSA]; UT, untransfected; Ctrl #1, siControl ON-TARGETplus Non-targeting siRNA #1 (Dharmacon); Ctrl #2, firefly luciferase-targeting siRNA; ZEB1 siRNA #1/#2, ZEB1-targeting siRNAs. Ctrl #2 and ZEB1 siRNA sequences are provided in Additional file 1 (Table S3). Chinese Hamster Ovary cells stably expressing human CAR (CHO+), or vector (CHO-) [9]. Loading controls are shown as β-actin, β-tubulin, GAPDH and PARP signals, with GAPDH as a cytoplasmic, and PARP as a nuclear marker.

We sought to investigate whether ZEB1 also contributes to the repression of CAR in PANC-1 cells in the context of TGF-β-mediated EMT, and whether it mediates CAR repression in established mesenchymal MDA-MB-231 cells. TGF-β reduces both CAR and E-cadherin protein levels in the absence but not in the presence of ZEB1 siRNA suggesting that the TGF-β-induced repression of either protein requires ZEB1 (Figure 5A). Similarly, ZEB1 plays a pivotal role in maintaining mesenchymal characteristics of MDA-MB-231 cells, since siRNA-mediated knockdown of ZEB1 induces a partial MET, illustrated by the up-regulation of epithelial markers such as CAR and E-cadherin, or the down-regulation of the mesenchymal marker fibronectin (Figure 5A and [34]). Interestingly, even though both siRNAs reduced ZEB1 protein levels similarly, transfection of PANC-1 cells with siRNA #2 down-regulated phospho-Smad2 (Figure 5A). Since ZEB1 siRNA #2 has a seed region that is 100% complementary to a region within the 3'UTR of phosphoinositide-3-kinase, regulatory subunit 1 (alpha) (PIK3R1, Entrez Gene, Gene ID: 5295, NCBI) [51], the effect on Smad2 might have been a consequence of reduced PI3K activity. The requirement of PI3K signaling for TGF-β1-mediated C-terminal phosphorylation of Smad2 was previously demonstrated in NMuMG cells [52].

### TGF-β does not affect ZEB1 protein levels or subcellular localization

While TGF-β only minimally up-regulated ZEB1 mRNA in PANC-1 cells (Figure 3D and Additional file 1, Fig. S1), effects at the protein level varied: some (Figure 5A) but not all (Figure 5B) experiments suggested that stimulation by TGF-β increases the total ZEB1 protein levels. To address this question systematically, we measured ZEB1 protein levels over time, with harvests of the total protein fractions in twenty-four hour intervals. Indeed, while CAR was down-regulated at every time point in the TGF-β-treated samples, ZEB1 levels remained unchanged throughout the time-course (Figure 5B). To investigate whether TGF-β promotes nuclear entry of ZEB1 as a mechanism to increase the latter protein's activity as a transcriptional repressor of CAR, we measured ZEB1 protein levels in both nuclear and cytoplasmic fractions. Interestingly, ZEB1 appears to be exclusively localized in the nucleus, both in the presence and absence of TGF-β. In agreement with the total ZEB1 protein data, TGF-β stimulation for forty-eight hours did not increase the nuclear ZEB1 levels (Figure 5C).

### ZEB1 is necessary for TGF-β-induced EMT in PANC-1 cells

**Figure 6:**
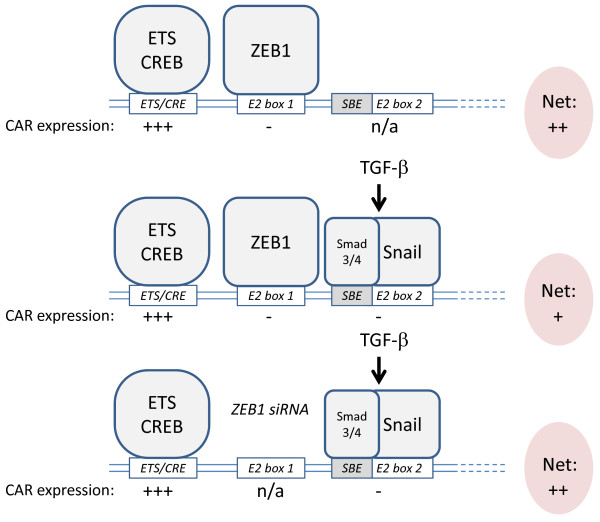
**ZEB1 as a constitutive repressor of the CAR promoter in PANC-1 cells**. Our data supports a model in which ZEB1 constitutively binds to E2 box 1 (Fig. 4B) in the human CAR promoter thereby controlling the basal levels of CAR. TGF-β may further repress the promoter via E2 box 2 and its adjacent Smad-binding element (*SBE*) by a mechanism likely involving Snail-Smad3/4 [37]. Knockdown of ZEB1 increases CAR expression despite the presence of Snail-Smad3/4. Factors binding to the *ETS-CRE *region (Fig. 1C), here speculatively indicated as ETS or CREB, strongly induce CAR expression ("+++"), while ZEB1 and Snail-Smad3/4 negatively ("-") regulate the CAR promoter. Net CAR expression indicates overall promoter activity. See text for details.

As demonstrated above, ZEB1 total, nuclear and cytoplasmic protein levels were little affected by TGF-β, whereas knockdown experiments suggested that ZEB1 is a critical component of the TGF-β-induced EMT process in PANC-1 cells (Figure 5A-B). To address this dilemma, we tested the hypothesis that TGF-β can *activate *ZEB1 rather than increase its protein levels. However, in reporter assays carried out with PANC-1 cells, TGF-β did not appear to enhance the repressor effect of overexpressed ZEB1 on the CAR promoter (data not shown). Still, even though this data does not support our hypothesis, the real effect of TGF-β on ZEB1 may have been masked as ZEB1 was likely highly overexpressed (Additional file 1, Fig. S1). Alternatively, our data is consistent with a model in which ZEB1 constitutively binds to one of the two E2 boxes (E2 box 1, Figure 4B) in the CAR promoter thereby controlling the basal levels of CAR. TGF-β may further repress the CAR promoter via the second E2 box (E2 box 2), for instance by activating Snail-Smad3/4 [37] (Figure 6).

### ZEB1 knockdown facilitates adenovirus uptake

**Figure 7:**
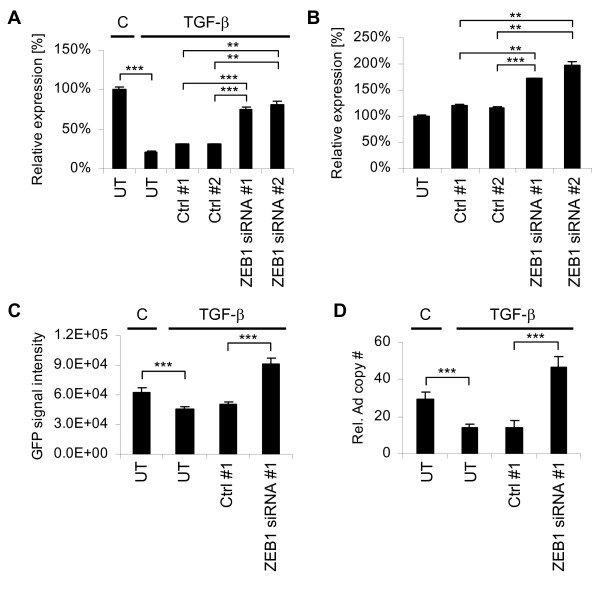
**Up-regulation of cell-surface CAR levels upon ZEB1 knockdown coincides with increased adenoviral infectability**. **A **and **B**. Cell-surface CAR levels following ZEB1 knockdown were measured by flow cytometry on PANC-1 cells treated with TGF-β1 (**A**), or on MDA-MB-231 cells (**B**). Error bars represent standard deviations (biological duplicates). **C **and **D**. Ad-GFP uptake following ZEB1 knockdown was determined by flow cytometry measuring GFP levels (**C**), or by real-time PCR for virus copy number (**D**). Error bars represent standard deviations. **A-D**. CAR and GFP signals are expressed as geometric means of the fluorescence signal intensities normalized to the untreated controls (A-C), virus copy numbers as relative adenovirus fiber levels (D). Abbreviations: UT, untransfected; Ctrl #1, siControl ON-TARGETplus Non-targeting siRNA #1 (Dharmacon); Ctrl #2, firefly luciferase-targeting siRNA; ZEB1 siRNA #1/#2, ZEB1-targeting siRNAs. Ctrl #2 and ZEB1 siRNA sequences are provided in Additional file 1 (Table S3). p < 0.001 (***), p < 0.01 (**) (Student's *t*-test, 1-tailed, equal variance).

An increase in CAR expression following ZEB1 knockdown may improve therapies with oncolytic adenoviruses if it translates into elevated cell-surface CAR levels [3]. We addressed this question in both PANC-1 EMT, and MDA-MB-231 MET models. In the former system, we employed the strategy outlined above, i.e. knockdown of ZEB1 in combination with TGF-β treatment. Consistent with the Western blot data (Figure 5A), ZEB1 knockdown indeed antagonized the TGF-β-induced reduction of the cell-surface CAR levels measured by flow cytometry (Figure 7A). Analogously, silencing of ZEB1 in MDA-MB-231 cells increased cell surface CAR expression (Figure 7B).

In agreement with the total CAR protein (Figure 5A) and cell-surface CAR (Figure 7A) data, PANC-1 cells with silenced ZEB1 expression were more susceptible to infection with a green fluorescence protein (GFP)-encoding adenovirus (Ad-GFP) [9] than the TGF-β treated non-silencing controls. This effect was apparent both at the level of GFP signal intensity (Figure 7C) and virus copy number (Figure 7D). For both methods, cells were harvested twenty-four hours post infection and were either analyzed by flow cytometry (GFP signal) or by TaqMan PCR (copy number) using adenoviral DNA (extracted together with cellular genomic DNA) as template. This data indicates that knockdown of ZEB1 might be a suitable approach to improve cellular uptake of therapeutic adenoviruses.

## Discussion

Up-regulation of CAR may be achieved by treatment with pharmacological inhibitors of RAS-MEK [5], of TGF-β signaling [9], or with HDAC inhibitors [6,7]. Here, we have demonstrated that ZEB1 plays a prominent role in the TGF-β-induced down-regulation of CAR, and that knockdown of ZEB1 is sufficient to improve adenovirus uptake.

We have previously noticed similar expression patterns for CAR and E-cadherin, and thus hypothesized that the underlying regulatory mechanisms are related. Here, we have functionally defined the minimal human CAR promoter and have shown that it contains four orthologously conserved motifs: putative *ETS *and *CRE *elements, and two closely spaced E2 boxes. Particularly the latter elements caught our attention, since they were reported to interact with E2 box transcriptional repressors such as ZEB1 [25,33-36] and SIP1 [23,33,35,39] in the E-cadherin promoter. Furthermore, the genetic context of the E2 boxes in the CAR (Figure 1B) and E-cadherin [39] promoters is similar. Indeed, overexpressed ZEB1 repressed the activity of the -291/-1 CAR promoter, and bound to CAR promoter oligonucleotides and chromatin. It is of note that Pong *et al*. suggested that the functional CAR promoter is located between -585 and -400 [10]. However, since the latter study did not address the role of the E2 boxes and primarily focused on CAR upstream sequence mediating positive regulation of promoter activity, it does not contradict our findings. Indeed, we have shown that the -681/-1 CAR upstream fragment, containing the proposed -585/-400 promoter, is associated with high promoter activity (Figure 1A).

Our ZEB1 knockdown experiments provide evidence that ZEB1 is a physiological repressor of CAR expression in PANC-1 and MDA-MB-231 cells. However, even though knockdown of ZEB1 was sufficient to antagonize the TGF-β-induced down-regulation of CAR and E-cadherin (Figure 5A), we did not observe consistent changes of the ZEB1 protein levels in PANC-1 cells neither in total nor nuclear fractions as consequence of the TGF-β stimulation (Figure 5A-C). Therefore, in our PANC-1 EMT model, TGF-β may *activate *ZEB1 rather than *up-regulate *its expression. Underlying mechanisms have not been described yet but may include posttranslational modification of ZEB1 or physical binding to TGF-β downstream effectors. For instance, TGF-β may enhance ZEB1's repressor activity by up-regulating expression and/or activity of ZEB1-associated co-repressors such as CtBP-1/-2 and/or BRG1. In support, TGF-β stimulation increased both *ctbp1 *and *brg1 *mRNA levels in NMuMG cells ([53], supplementary table I), a murine cell line for which we and others reported a TGF-β-mediated down-regulation of CAR [9,37]. However, in contrast to our data obtained with (human) PANC-1 cells, NMuMG cells responded to TGF-β stimulation with increased ZEB1 (δEF1) expression [35]. Nevertheless, BRG1 was shown to physically associate with ZEB1 to repress the E-cadherin promoter [54].

Even though ZEB1 is necessary for the TGF-β-induced inhibition of CAR expression, TGF-β may activate factors other than co-repressors that physically interact with ZEB1 to down-regulate CAR. In such a model, ZEB1 would play a role as a constitutive repressor of CAR and thereby counteract activating factors such as those interacting with the *ETS *and *CRE *elements (Figure 1). siRNA-mediated depletion of ZEB1 would ease repression and consequentially increase CAR levels. Such a model appears attractive (Figure 6): Snail-Smad3/4 was shown to repress the mouse CAR promoter by a mechanism that involves interactions with E2 boxes and adjacent Smad-binding elements (*SBE*s) [37]. Intriguingly, similarly to the mouse CAR promoter, E2 box 2 in the human CAR promoter contains an adjacent *SBE *(5'-CAGA-3') as well (Figure 1B). This may indicate that the human CAR promoter can also potentially be inhibited by Snail-Smad3/4 [37]. Therefore, ZEB1 may regulate the basal CAR levels by mediating a certain degree of promoter inhibition when bound to E2 box 1. However, further repression through binding of Snail-Smad3/4 to E2 box 2 may occur upon stimulation with TGF-β (Figure 6). The assumption that the mesenchymal factor ZEB1 is bound to the CAR promoter even in the absence of TGF-β may be regarded as a discrepancy to the epithelial features of PANC-1 cells (Figure 2, 3 and 5). However, even though these cells undergo TGF-β-induced EMT, they may not be prototypical epithelial cells as they express some mesenchymal/stem cell markers and can be brought into a more typical epithelial state by inhibiting Cyr61 [55]. Furthermore, even though functional characterization of the role of Snail-Smad3/4 on the CAR promoter was conducted in mouse cells, in invasive human ductal breast carcinoma, nuclear expression of Snail, Smad3 and Smad4 correlated with loss of CAR expression at the invasive front [37]. This data is consistent with our model which postulates that Snail-Smad3/4 may also negatively regulate the human CAR promoter (Figure 6).

Our work identifies ZEB1 as a negative regulator of cell-surface CAR expression and adenovirus uptake and thus as a candidate therapeutic target in treatment strategies with oncolytic adenoviruses. Responsive tumor types may include moderately to poorly differentiated gastrointestinal tumors with low CAR expression [4]. However, whether or not this approach is successful does not solely depend on how efficiently the virus is taken up by the respective target cells, but also how effectively it replicates once taken up. We and others recently demonstrated that p21^WAF1 ^acts as a negative regulator of adenovirus replication [7,11]. For instance, even though the HDAC inhibitor valproic acid (VPA) up-regulated CAR, and facilitated adenovirus uptake, it additionally increased p21^WAF1 ^levels and reduced virus replication [7]. Therefore, if such a scenario also applies to approaches targeting ZEB1, it might be necessary to engineer a replication-competent adenovirus able to silence p21 expression to improve replication and cell killing.

In summary, we have shown that ZEB1 negatively regulates CAR expression and adenovirus uptake in the context of TGF-β-mediated EMT, and that inactivation of ZEB1 may induce some form and degree of MET. We have demonstrated that knockdown of ZEB1 antagonized the TGF-β-mediated EMT process and the down-regulation of CAR in PANC-1 cells.

## Conclusions

Our findings may suggest that carcinoma cells *in vivo*, stimulated by stroma-derived TGF-β, might respond to ZEB1 inactivation with MET resulting in reduced invasiveness and CAR up-regulation, and in improved adenovirus uptake. The latter effect may translate into more effective therapies utilizing oncolytic adenoviruses.

## Competing interests

The authors declare that they have no competing interests.

## Authors' contributions

MDL designed and carried out experiments, and participated in the writing of the manuscript. MS conducted RNAi, adenoviral and immunofluorescence experiments. MIT performed RNAi and adenoviral experiments, PC was involved in construct generation and carrying out immunoblotting experiments. DK aligned orthologous CAR promoter sequences obtained from GenBank and Ensembl databases. WMK conceived and oversaw the project, and participated in the writing of the manuscript. All authors reviewed and approved the final manuscript.

## Supplementary Material

Additional file 1 **Figure S1, Tables S1-S3, and supplemental methods**. **Figure S1 **demonstrates effects of TGF-β and overexpressed ZEB1 on *SERPINE 1 *(PAI-1) and *CXADR *(CAR) mRNA expression in PANC-1 cells. **Table S1 **contains corresponding GenBank (NCBI) and Ensemble database accession numbers and positions of the translational start ATG of the orthologous CAR promoter sequences used for the alignment shown in Figure 1B. **Tables S2 and S3 **contain oligonucleotide sequences. **Supplemental methods **include additional experimental procedures and in-depth descriptions of methods outlined above. Click here for file
