# Supplementary material for: ZEB1 limits adenoviral infectability by transcriptionally repressing the Coxsackie virus and Adenovirus Receptor
Source: Mol Cancer. 2011 Jul 27;10:91. doi: 10.1186/1476-4598-10-91 (PMC3164624; doi:10.1186/1476-4598-10-91)
Supplement: Additional file 1 — Figure S1, Tables S1-S3, and supplemental methods. Figure S1 demonstrates effects of TGF-β and overexpressed ZEB1 on SERPINE 1 (PAI-1) and CXADR (CAR) mRNA expression in PANC-1 cells. Table S1 contains corresponding GenBank (NCBI) and Ensemble database accession numbers and positions of the translational start ATG of the orthologous CAR promoter sequences used for the alignment shown in Figure 1B. Tables S2 and S3 contain oligonucleotide sequences. Supplemental methods include additional experimental procedures and in-depth descriptions of methods outlined above. [file 1476-4598-10-91-S1.DOCX]

**Supplemental information (Additional file 1, Lacher *et al.*)**

**Figure S1. Overexpression of ZEB1.** PANC-1 cells were transiently transfected with an inducible full-length Myc-ZEB1 expression construct, and a “Tet-OFF” plasmid allowing induction of *ZEB1* by absence (-Dox), and repression by addition (+Dox) of doxycycline to the culture medium. After transfection, cells were stimulated with TGF-β1 for 2 days prior to lysis for RNA extraction, reverse transcription and TaqMan real-time PCR with primer/probe sequences listed in Table S3, or with Expression Assays (ABI) indicated under “Expression analysis by real-time RT-PCR” in the main manuscript. Data was normalized to averaged *H3F3A* (H3 histone, family 3A) and *GUSB* mRNA levels (arbitrary units). *SERPINE1* (PAI-1) is a known TGF-β target gene and serves as positive control for the biological activity of the TGF-β used [[1](#_ENREF_1)]. Error bars represent standard deviations (biological triplicates). UT = untransfected.

**Materials and Methods:**

**Constructs.**

The pGL3-Enhancer luciferase vector (Promega, Madison, WI, USA) was made Gateway-compatible (Invitrogen, Carlsbad, CA, USA) by inserting the *att*R1-[Cmr-*ccd*B]-*att*R2 cassette (reading frame A; Invitrogen) and inserted into the blunt-ended *Hind*III restriction site, resulting in the pGL3En-DESTfw/HindIII construct. A *Lox*P-encoding, KpnI/SalI-digested PCR product, obtained through extension of the overhangs of the annealed LoxP F/R (Table S2) oligonucleotides, was cloned into the *Kpn*I site of pGL3En-DESTfw/HindIII, resulting in pGL3En-DESLoxX. The *Kpn*I-{*Lox*P- *att*R1-[Cmr-*ccd*B]-*att*R2 cassette}-*Nar*I fragment was then transferred into the KpnI/NarI-digested pGL3-Basic vector (Promega, Madison, WI, USA), resulting in pGL3Ba-DESLoxX. Introduction of the selectable *neo*^R^ marker was achieved through Cre-mediated homologous recombination of the pExchange module EC-Neo (Stratagene, La Jolla, CA, USA) into the *Lox*P site of pGL3Ba-DESLoxX, resulting in pGL3Ba-DESneo. *Nco*I sites not encoding the luciferase translational start ATG codon, or located in the *att*R1-[Cmr-*ccd*B]-*att*R2 cassette, were removed through sequential site-directed mutagenesis with the NcoI-1 F/R, NcoI-2 F/R, and NcoI-3 F/R (Table S2) primer pairs, resulting in pGL3Ba-DESneo3N. Sequencing revealed an inconsequential incorporation of 41 bp of the NcoI-3F/NcoI-3R primer pair at the NcoI-3 region.

The pGL3Ba-DESneo3N vector allows rapid cloning of [promoter]-[5’-UTR] fragments in endogenous constellation, i.e. with no vector sequence between the 3’ end of the 5’-UTR (untranslated region) and the translational start ATG of the firefly luciferase gene. Cloning is dependent on the presence of an *Nco*I restriction site (CCATGG) at the translational start ATG, a feature expected to be found in a variety of genes since *Nco*I sites are present in Kozak elements [[2](#_ENREF_2)]. CAR promoter fragments were independently PCR-amplified from human genomic DNA with the hMin2017, hMin1195, hMin926, hMin890, hMin681, hMin291, and hMin96 forward primers, each in combination with the hPlus41 reverse primer (Table S2). Reactions were carried out with the high-fidelity Platinum^®^ or AccuPrime^TM^ *Pfx* DNA polymerases (both Invitrogen). PCR fragments were cloned into pENTR/D-TOPO^®^ (Invitrogen), then transferred into pGL3Ba-DESneo3N via homologous recombination (Gateway technology, Invitrogen). Sequence between the translational ATG start codons of CAR and luciferase was removed by NcoI/AscI digestion, followed by ethanol precipitation and re-ligation of the *Nco*I-*Nco*I fragments. To obtain the pGL3Ba-DESneo3N-EmVec “Empty Vector” plasmid, first the CAR promoter/5’-UTR fragment from one of the pGL3Ba-DESneo3N-derived constructs was excised with SacII and NcoI, then, after Klenow enzyme-catalyzed blunt-end generation and ethanol precipitation, the vector was re-ligated with T4 DNA ligase. Mutations in E2 boxes, *ETS* and cAMP responsive element (*CRE*) motifs were introduced by site-directed mutagenesis using the -291/-1 luciferase construct as template in combination with primer pairs E2bx1 F/R, E2bx2 F/R, or E2bx1and2 F/R (E2 boxes), or ETS F/R and CRE F/R, respectively. Primer sequences are provided in Table S2.

To generate inducible Myc-tagged ZEB1 expression constructs, the mSIP1 coding sequence (cds) between the *Sal*I and *Xba*I sites on pUHD10.3SIP1 [[3](#_ENREF_3)] was replaced by an AccuPrime^TM^ *Pfx* DNA Polymerase-amplified *Sal*I-*Xba*I fragment containing human *ZEB1* cds, resulting in pTRE-6Myc-ATGhZEB1. PCR was carried out with the hZEB1-ATG and hZEB1-TAA primers (Table S2) and plasmid-encoded *ZEB1* (Open Biosystems, Huntsville, AL, USA) as template. To avoid a potential translation downstream of the N-terminal Myc tags, the translational start codon of ZEB1 in pTRE-6Myc-ATGhZEB1 was destroyed via site-directed mutagenesis using primers hZEB1-N2 F/R (Table S2), resulting in pTRE-6Myc-deltaATG-hZEB1. To obtain ZEB1 with an N-terminal deletion of 65 amino acids, a *Sal*I restriction site was introduced at the corresponding position of the ZEB1 cds in pTRE-6Myc-ATGhZEB1, and a *Sal*I-*Sal*I fragment was removed.

Restriction enzymes, T4 DNA ligase and Klenow enzyme were purchased from New England Biolabs (Ipswich, MA, USA). Site-directed mutagenesis was carried out with the QuikChange^TM^ (pGL3Ba-DESneo3N) or QuikChange® II (all other constructs) XL Site-Directed Mutagenesis kits (Stratagene). Primer design was assisted by Primer 3 [[4](#_ENREF_4)] or Primer X (mutagenesis primers) [[5](#_ENREF_5)] software.

**RNA interference.** Experiments were conducted with cells plated at low density (20-40% confluency at the beginning of TGF-β or siRNA stimulation). PANC-1 cells were pre-treated for two days with 5 ng/mL platelet-derived human TGF-β1 (R&D Systems, Minneapolis, MN, USA), then, and two days later, siRNA-transfected by using the Lipofectamine RNAiMax transfection reagent (Invitrogen) with 3.0 microliter (6-well format) or 1.2 microliter (12-well format) per well according to the manufacturer’s recommendations. TGF-β treatment was continued through the first transfection until two days after the second one. MDA-MB-231 cells were similarly transfected, except that 6.0 microliter of the transfection reagent were used per well (6-well format). Cell lysis for protein harvest, flow cytometric analysis of cell-surface CAR and adenovirus infections were carried out four days after the initial transfection. All siRNAs were synthesized with ON-TARGETplus modifications (Dharmacon/Thermo Fisher Scientific, Inc., Rockford, IL, USA) and used at 20 nM. Ctrl #2 and both ZEB1 siRNAs were designed with the si*DESIGN*^®^ Center tool (Dharmacon). Abbreviations: UT, untransfected; Ctrl #1, siControl ON-TARGETplus Non-targeting siRNA #1 (Dharmacon); Ctrl #2, firefly luciferase-targeting siRNA; ZEB1 siRNA #1/#2, ZEB1-targeting siRNAs. Ctrl #2 and ZEB1 siRNA sequences are provided in Table S3 and were obtained by using the si*DESIGN*^®^ Center (Dharmacon/Thermo Fisher Scientific, Inc.).

**Biotinylated Oligonucleotide Precipitation Assay.** One day after seeding 3x10^6^ PANC-1 cells per 10 cm-dish, cells were transiently co-transfected with pRevTet-Off (4.0 microgram) (Clontech Laboratories, Inc./Takara Bio, Inc., Otsu, Shiga, Japan) in combination with pTRE-6Myc-deltaATG-hZEB1 (12.0 microgram) by using FuGENE HD (Roche, Indianapolis, IN, USA) (3 microliter per 1 microgram of DNA). Control lysates were made from PANC-1 cells seeded at a density of 5x10^5^ cells per well (6-well plate) and transfected with the same plasmids. Four hours post transfection, transfection medium was replaced by antibiotic-containing full medium. Six hours post transfection, medium was again replaced by full medium with (to repress ZEB1) or without (to induce ZEB1) 2 microgram/mL doxycycline hyclate (Sigma-Aldrich, St. Louis, MO, USA). Forty-eight hours after transfection, oligonucleotide precipitations were carried out following a modified version of the procedure described by others [[3](#_ENREF_3), [6](#_ENREF_6)].

In essence, a whole cell protein extract was prepared from cells lysed in 1500 microliter/10 cm-dish or 300 microliter/well RIPA buffer (Sigma-Aldrich) containing protease inhibitors (Complete Mini, Roche) and Phosphatase Inhibitor Cocktail I (Sigma-Aldrich). Per sample, 250 microgram of protein (ZEB1 induced) in 150 microliter RIPA buffer and 250 microliter HKMG buffer [[6](#_ENREF_6)] containing protease and phosphatase inhibitors (as above) were supplemented with 150 microliter 50% agarose-streptavidin beads (Streptavidin Agarose Resin; Pierce/Thermo Fisher Scientific, Inc., Rockford, IL, USA) and pre-cleared for 90 minutes by rotating at 4°C. 475 microliter of the pre-cleared protein extract (representative of 250 microgram crude protein) were supplemented with 10 microliter (1 microgram) PAGE-purified 5’-biotinylated CAR or E-cadherin promoter oligonucleotide (Integrated DNA Technologies, Inc., Coralville, IA, USA; Table S3; sequences for the E-cadherin promoter controls are shown in Table S3 [4915-4986 of NG_008021.1 (RefSeqGene, NCBI) without the “C” at 4962, as shown in Figure 2E in [[3](#_ENREF_3)]] and 15 microliter (15 microgram) poly(dI-dC).poly(dI-dC) (Sigma-Aldrich) and rotated for 16 hours at 4°C. ZEB1-DNA complexes were pulled down with 25 microliter 50% agarose-strepavidin beads (as above) for approximately 90 minutes by rotating at 4°C. Beads were washed twice with HKMG buffer and ZEB1 protein was eluted at 95°C in 30 microliter 2X NuPAGE LDS sample buffer containing 2X NuPAGE reducing agent (both Invitrogen). 20 microliter per eluate were analyzed by immunoblotting as described [[7](#_ENREF_7)]. ZEB1 was detected with 1 microgram/mL of the mouse monoclonal anti-Myc Tag clone 4A6 (Upstate/Millipore, Charlottesville, VA, USA).

**Flow cytometry.** Cells were detached in 250 microliter/well (6-well plates) 0.05% trypsin containing 0.02% (w/v) EDTA [University of California (UCSF) Cell Culture Facility, CA, USA]. After addition of 800 microliter/well 100% FBS (Valley Biomedical, Inc.; Winchester, VA, USA), 600 microliter of each cell suspension were transferred into microtubes for CAR staining. For the isotype-matched control samples, 300 microliter of each remaining cell suspension were pooled with the corresponding biological duplicate sample (same siRNA condition). After 1X washing with 1 mL PBS (w/o Ca^2^+ and Mg^2^+) containing 0.04% (w/v) EDTA (UCSF Cell Culture Facility) supplemented with 5% FBS (as above) (P5F) cells were stained in 200 microliter P5F containing 2 microgram phycoerythrin (PE)-conjugated control IgG-PE (mouse monoclonal IgG1 κ, Pharmingen/BD Biosciences, San Jose, CA, USA) or anti-CAR-PE antibody (E1-1, mouse monoclonal; Santa Cruz Biotechnology, Inc., Santa Cruz, CA, USA) while rotating for 60 minutes at 4°C. Cells were then washed 1X as above, resuspended in 1 micromolar TO-PRO^®^-3 iodide (TP3) (Invitrogen) in P5F, and analyzed by flow cytometry on a FACSCalibur (BD Biosciences) or an Accuri C6 flow cytometer (Accuri Cytometers, Inc., Ann Arbor, MI, USA/BD, Franklin Lakes, NJ, USA). Cell-surface CAR was detected in the FL2 channel, non-viable cells, stained by TP3 and detected in the FL4 channel, were excluded. For the analysis of live Ad-GFP infected cells, GFP was detected in the FL1 channel. TP3-positive cells were excluded. Data were analyzed with Cyflogic^TM^ software (CyFlo Ltd, Turku, Finland).

**Tables:**

ALIGNMENT OF ORTHOLOGOUS CAR UPSTREAM SEQUENCES

| **Species** | **Reference** | | | **Position ATG** | **Ori** |
| --- | --- | --- | --- | --- | --- |
|  | Accession #/Scaffold | GI | DB |  |  |
| Human (*Homo sapiens*) | NT_011512.10 | 51475294 | NCBI | 4547319 | + |
| Chimpanzee (*Pan troglodytes*) | NT_106996.1 | 52326515 | NCBI | 3977761 | + |
| Mouse (*Mus musculus*) | NT_039625.7 | 149268225 | NCBI | 13007154 | + |
| Rat (*Rattus norvegicus*) | NW_047354.2 | 62657745 | NCBI | 17193576 | + |
| Dog (Canis familiaris) | NW_876295.1 | 74001570 | NCBI | 14031230 | + |
|  | EU744539.1 | 189364823 | NCBI | 1 | + |
| Cattle (*Bos taurus*) | NW_001493756.1 | 119876895 | NCBI | 1924565 | + |
| Cat (*Felis catus*) | AANG01242110.1 | 94058441 | NCBI | 3407 | + |
| Chicken (*Gallus gallus*) | NW_001471532.1 | 118083772 | NCBI | 2506118 | + |
| Frog (*Xenopus tropicalis*) | scaffold_694 | N/A | Ensembl | 83618 | - |
| Zebrafish (*Danio rerio*) | NC_007121.3 | 189908151 | NCBI | 21102184 | + |

**Table S1. Orthologous CAR upstream sequences.** Chr, chromosome; GI, identifier in the GenBank (NCBI) or Ensemble database (release 52 - Dec 2008); DB, database; Ori, orientation of *CXADR* (CAR) gene on reference sequence.

| **Name** |  | **Sequence** | |
| --- | --- | --- | --- |
|  |  |  | |
| ***Construction of pGL3Ba-DESneo3N*** | | | |
| LoxP | F | 5’-ATGCGGTACCGGGCCCATGGATAACTTCGTATAGCATACATTATACGAAG-3’ | |
|  | R | 5’-TACGGTCGACTTAAAAGCTTAGGCCGGGCCATAACTTCGTATAATGTATGCTATACGAAG-3’ | |
| NcoI-1 | F | 5’-CGATAGGTACCGGGCCCATcGATAACTTCGTATAGCATAC-3’ | |
|  | R | 5’-GTATGCTATACGAAGTTATCgATGGGCCCGGTACCTATCG-3’ | |
| NcoI-2 | F | 5’-CAAGCAGGCATCGCCgTGGGTCACGACGAG-3’ | |
|  | R | 5’-CTCGTCGTGACCCAcGGCGATGCCTGCTTG-3’ | |
| NcoI-3 | F | 5’-CATAAATAAAAAAAATTAGTCAGCCtTGGGGCGGAGAATGGGCGGAACTG-3’ | |
|  | R | 5’-CAGTTCCGCCCATTCTCCGCCCCAaGGCTGACTAATTTTTTTTATTTATG-3’ | |
|  |  |  | |
| ***Construction of human CAR promoter/5’-UTR reporter systems*** | | | |
| hMin2017 | F | 5’-CACCGGGAAGTCCCTAGCCGTTAC-3’ | |
| hMin1195 | F | 5’-CACCGCAGGAACCCTGTAAGCAAG-3’ | |
| hMin926 | F | 5’-CACCGAGCAGCCGACAACAACTTT-3’ | |
| hMin890 | F | 5’-CACCGACCCCACACTCCAACTGAC-3’ | |
| hMin681 | F | 5’-CACCGAGGGGCTCTATCCCTACCA-3’ | |
| hMin291 | F | 5’-CACCGGGAGGCTGAGAGTTC-3’ | |
| hMin96 | F | 5’-CACCGCGAGCCAGTCGGGA-3’ | |
| hPlus41 | R | 5’-ACTACTCCGCACAGGAGCAC-3’ | |
| E2bx1 | F | 5’-GGAAGTGACGCGAGTTCAtCTGCCGAGCG-3’ | |
|  | R | 5’-CGCTCGGCAGaTGAACTCGCGTCACTTCC-3’ | |
| E2bx2 | F | 5’-AGGGTGCAGAGaTGCCGCCGCCGCCGCGAG-3’ | |
|  | R | 5’-CTCGCGGCGGCGGCGGCAtCTCTGCACCCT-3’ | |
| E2bx1and2 | F | GGAAGTGACGCGAGTTCAtCTGCCGAGCGGGGGCTGGGAGGAGGGGCGGAGGGTGCAGAGaTGCCGCCGCCGCCGCGAG | |
|  | R | CTCGCGGCGGCGGCGGCAtCTCTGCACCCTCCGCCCCTCCTCCCAGCCCCCGCTCGGCAGaTGAACTCGCGTCACTTCC | |
| ETS | F/R | 5’-CCCGGGCCGCTGtgaGAAGTGACGCGAG-3’/5’-CTCGCGTCACTTCtcaCAGCGGCCCGGG-3’ | |
| CRE | F/R | 5’-CTGCCGGAAGTGAatCGAGTTCACCTGCC-3’/5’-GGCAGGTGAACTCGatTCACTTCCGGCAG-3’ | |
|  |  |  | |
| ***Construction of pTRE-6Myc-deltaATG-hZEB1*** | | | |
| hZEB1-ATG | F | | 5’-AGCGAGtcGAcCATGGCGGATGGCCCCAGGTG-3’ |
| hZEB1-TAA | R | | 5’-TTCCTTCTAGAAAAACGATTAGGCTTC-3’ |
| hZEB1-N2 | F/R | | 5’-GATCTCGAATTCAAGGTCGACgtcGaCGGATGGCCCC-3’/5’-GGGGCCATCCGtCgacGTCGACCTTGAATTCGAGATC-3’ |

**Table S2. Oligonucleotides.** Primers used for cloning purposes. Underscored small letters indicate mismatches to the wild-type sequence. PCR primers were purchased from Invitrogen. F, forward; R, reverse.

| **Name** |  | **Sequence** |
| --- | --- | --- |
|  |  |  |
| ***siRNA sequences*** | | |
| Ctrl #2 | S/AS | 5’-GGAAAGACGAUGACGGAAAUU-3’/5’-UUUCCGUCAUCGUCUUUCCUU-3’ |
| ZEB1 siRNA #1 | S/AS | 5’-CAGUGAAAGAGAAGGGAAUUU-3’/5’-AUUCCCUUCUCUUUCACUGUU-3’ |
| ZEB1 siRNA #2 | S/AS | 5’-AACUGAACCUGUGGAUUAUUU-3’/5’-AUAAUCCACAGGUUCAGUUUU-3’ |
|  |  |  |
| ***Real-time PCR primers and probes*** | | |
| *mRNA Expression (TaqMan PCR)* | | |
| CAR [[8](#_ENREF_8)] | F/R | 5’-GGCGCTCCTGCTGTGC-3’/5’-CTTTGGCTTTTTCAATCATCTCTTC-3’ |
|  | Pr | 5’-(6FAM)-TGCGGAGTAGTGGATTTCGCCAGAAG-(TAMRA)-3’ |
| H3F3A | F/R | 5’-GCTTCCAGAGCGCAGCTATC-3’/5’-GGCGTGCTAGCTGGATGTCT-3’ |
|  | Pr | 5’-(6FAM)-TGCTTTGCAGGAGGCAAGTGAGGC-(TAMRA)-3’ |
| GUSB | F/R | 5’-CTCATTTGGAATTTTGCCGATT-3’/5’-CCGAGTGAAGATCCCCTTTTTA-3’ |
|  | Pr | 5’-TGAACAGTCACCGACGAGAGTGCTGG-3’ |
| *mRNA Expression (SYBR Green PCR)* | | |
| UniSTS #34015  *(for endog. ZEB1)* | F | 5’-CCTTGCTTTTCATGGAAACA-3’ (http://www.ncbi.nlm.nih.gov/genome/sts/sts.cgi?uid=34015) |
|  | R | 5’-GGCACACCCGGATTTA-3’ (http://www.ncbi.nlm.nih.gov/genome/sts/sts.cgi?uid=34015) |
| HuZEB1-CDS [[9](#_ENREF_9)]  *(for total ZEB1)* | F | 5’-AGCAGTGAAAGAGAAGGGAATGC-3’ |
|  | R | 5’-GGTCCTCTTCAGGTGCCTCAG-3’ |
| GUSB | F | 5’-CTCATTTGGAATTTTGCCGATT-3’ |
|  | R | 5’-CCGAGTGAAGATCCCCTTTTTA-3’ |
| *Chromatin Immunoprecipitation (ChIP)* | | |
| CAR | F | 5’-GCCGCTGCCGGAAGTGACGCGA-3’ |
|  | R | 5’-CTGCAGGTAGGCGGCTCT-3’ |
| *Ad copy #* | | |
| Ad fiber | F/R | 5’-CATGTTGTTGCAGATGAAGCG-3’/5’-GGCACAGTTGGAGGACCG-3’ |
|  | Pr | 5'-(6FAM)-AACCCCGTGTATCCATATGACACGGAAA-(TAMRA)-3' |
|  |  |  |
| ***Biotinylated Oligonucleotide Precipitation Assay*** | | |
| hEcadWT | F | 5’-GGCCGGCAGGTGAACCCTCAGCCAATCAGCGGTACGGGGGGCGGTGCTCCGGGGCTCACCTGGCTGCAGCC-3’ |
|  | R | 5’-GGCTGCAGCCAGGTGAGCCCCGGAGCACCGCCCCCCGTACCGCTGATTGGCTGAGGGTTCACCTGCCGGCC-3’ |
| hEcadBx1-3 | F | 5’-GGCCGGCAGaTGAACCCTCAGCCAATCAGCGGTACGGGGGGCGGTGCTCCGGGGCTCAtCTGGCTGCAGCC-3’ |
|  | R | 5’-GGCTGCAGCCAGaTGAGCCCCGGAGCACCGCCCCCCGTACCGCTGATTGGCTGAGGGTTCAtCTGCCGGCC-3’ |
| hCARwt | F | 5’-AGTGACGCGAGTTCACCTGCCGAGCGGGGGCTGGGAGGAGGGGCGGAGGGTGCAGAGGTGCCGCCGCCG-3’ |
|  | R | 5’-CGGCGGCGGCACCTCTGCACCCTCCGCCCCTCCTCCCAGCCCCCGCTCGGCAGGTGAACTCGCGTCACT-3’ |
| hCARbx1 | F | 5’-AGTGACGCGAGTTCAtCTGCCGAGCGGGGGCTGGGAGGAGGGGCGGAGGGTGCAGAGGTGCCGCCGCCG-3’ |
|  | R | 5’-CGGCGGCGGCACCTCTGCACCCTCCGCCCCTCCTCCCAGCCCCCGCTCGGCAGaTGAACTCGCGTCACT-3’ |
| hCARbx2 | F | 5’-AGTGACGCGAGTTCACCTGCCGAGCGGGGGCTGGGAGGAGGGGCGGAGGGTGCAGAGaTGCCGCCGCCG-3’ |
|  | R | 5’-CGGCGGCGGCAtCTCTGCACCCTCCGCCCCTCCTCCCAGCCCCCGCTCGGCAGGTGAACTCGCGTCACT-3’ |
| hCARbx1-2 | F | 5’-AGTGACGCGAGTTCAtCTGCCGAGCGGGGGCTGGGAGGAGGGGCGGAGGGTGCAGAGaTGCCGCCGCCG-3’ |
|  | R | 5’-CGGCGGCGGCAtCTCTGCACCCTCCGCCCCTCCTCCCAGCCCCCGCTCGGCAGaTGAACTCGCGTCACT-3’ |

**Table S3. Oligonucleotides.** Real-time PCR primers/probes and siRNA sequences. siRNAs (with ON-TARGETplus modifications) were purchased from Dharmacon, and biotinylated oligonucleotides from Integrated DNA Technologies, Inc. F, forward; R, reverse; S, sense; AS, antisense; Pr, probe.

**References:**

1. Murakami M, Ikeda T, Saito T, Ogawa K, Nishino Y, Nakaya K, Funaba M: **Transcriptional regulation of plasminogen activator inhibitor-1 by transforming growth factor-beta, activin A and microphthalmia-associated transcription factor.** *Cellular signalling* 2006, **18:**256-265.

2. Kozak M: **An analysis of 5'-noncoding sequences from 699 vertebrate messenger RNAs.** *Nucleic Acids Res* 1987, **15:**8125-8148.

3. Comijn J, Berx G, Vermassen P, Verschueren K, van Grunsven L, Bruyneel E, Mareel M, Huylebroeck D, van Roy F: **The two-handed E box binding zinc finger protein SIP1 downregulates E-cadherin and induces invasion.** *Mol Cell* 2001, **7:**1267-1278.

4. Rozen S, Skaletsky H: **Primer3 on the WWW for general users and for biologist programmers.** *In: Krawetz S, Misener S (eds) Bioinformatics Methods and Protocols: Methods in Molecular Biology, Humana Press, Totowa, NJ,* 2000, **132:**365-386.

5. Lapid C, Gao Y: **PrimerX - Automated design of mutagenic primers for site-directed mutagenesis.** [**http://www.bioinformatics.org/primerx/index.htm**](http://www.bioinformatics.org/primerx/index.htm)**.** [*http://wwwbioinformaticsorg/primerx/indexhtm*](http://wwwbioinformaticsorg/primerx/indexhtm) 2003.

6. Hata A, Seoane J, Lagna G, Montalvo E, Hemmati-Brivanlou A, Massague J: **OAZ uses distinct DNA- and protein-binding zinc fingers in separate BMP-Smad and Olf signaling pathways.** *Cell* 2000, **100:**229-240.

7. Lacher MD, Tiirikainen MI, Saunier EF, Christian C, Anders M, Oft M, Balmain A, Akhurst RJ, Korn WM: **Transforming growth factor-beta receptor inhibition enhances adenoviral infectability of carcinoma cells via up-regulation of Coxsackie and Adenovirus Receptor in conjunction with reversal of epithelial-mesenchymal transition.** *Cancer Res* 2006, **66:**1648-1657.

8. Anders M, Christian C, McMahon M, McCormick F, Korn WM: **Inhibition of the Raf/MEK/ERK pathway up-regulates expression of the coxsackievirus and adenovirus receptor in cancer cells.** *Cancer Res* 2003, **63:**2088-2095.

9. Clarhaut J, Gemmill RM, Potiron VA, Ait-Si-Ali S, Imbert J, Drabkin HA, Roche J: **ZEB-1, a repressor of the semaphorin 3F tumor suppressor gene in lung cancer cells.** *Neoplasia* 2009, **11:**157-166.
